# Rehabilitation in children with ventricular assist devices

**DOI:** 10.1016/j.jhlto.2025.100310

**Published:** 2025-05-31

**Authors:** Matthew J. O’Connor

**Affiliations:** Division of Cardiology, Children’s Hospital of Philadelphia, University of Pennsylvania Perelman School of Medicine, Philadelphia, Pennsylvania

**Keywords:** ventricular assist device, children, rehabilitation, exercise, nutrition, psychosocial health

## Abstract

Ventricular assist devices play a critical role in the management of end-stage heart failure, with a significant increase in their utilization in children over the past 20 years. While much attention has been given toward the success of ventricular assist devices in reversing the physiologic consequences of chronic heart failure, the ability of ventricular assist devices to facilitate rehabilitation should not be underestimated. For patients with chronic heart failure, rehabilitation can be understood to include exercise rehabilitation, nutritional rehabilitation, and psychosocial rehabilitation. In this review article, the effects of chronic heart failure on these 3 domains of rehabilitation are described, along with the impact of ventricular assist devices on these domains in children. Data demonstrating the effects of ventricular assist device therapy on rehabilitation are reviewed, and areas where opportunities for the improvement in care exist are identified as well.

The rapid adoption of ventricular assist devices (VAD) in children over the past 2 decades has had a major impact upon the care of children with heart failure (HF). Currently, approximately 40% of children undergoing heart transplantation (HT) have a VAD in place at the time of HT,^1^ with the proportion of patients supported with VAD nearly doubling during the first 2 decades of this millennium.^2^ The prevalence of children on VAD is only expected to rise given chronic HT donor availability constraints, along with the fact that VAD therapy is increasingly being offered to patients with congenital heart disease (CHD) and complex circulations.^3^, ^4^ VAD therapy reliably reverses the HF phenotype and can restore a near-normal quality of life (QOL) for many patients, particularly those who are large enough to undergo placement of a dischargeable device. VAD is also superior to medical therapy with respect to maximizing functional status in children awaiting transplantation.^5^ The most commonly utilized VADs in children are outlined in Table 1.

**Table 1:** Commonly Utilized Ventricular Assist Devices in Children

**Device type**	**Potential for hospital discharge**
Paracorporeal Continuous-Flow	
• Pedimag (Abbott) • Centrimag (Abbott) • Rotaflow (Getinge)	N N N
Paracorporeal Pulsatile-Flow	
• Berlin Heart EXCOR (Berlin Heart)^a^	N
Percutaneous Continuous-Flow	
• Impella (Abiomed)^b^	N
Intracorporeal Pulsatile-Flow	
• Syncardia Total Artificial Heart (Syncardia)	Y^c^
Intracorporeal Continuous-Flow	
• HeartWare (Medtronic)^d^ • HeartMate 3 (Abbott)	Y Y

Device type is listed with manufacturer in parentheses.

Of the above devices, the Berlin Heart, Impella, and HeartMate 3 devices are US Food and Drug Administration-approved for use in children.

a Available in 10 ml, 15 ml, 25 ml, 30 ml, 50 ml, and 60 ml pump sizes.

b Available in various catheter sizes with variable flow rates.

c With the use of the Freedom driving unit.

d Removed from the market in 2021.

Children with chronic HF are frequently sarcopenic, malnourished, and frail; as such, they are usually unfavorable candidates for HT as these characteristics are associated with inferior outcomes following HT.^6^, ^7^, ^8^ Multiple studies have demonstrated the benefit of VAD in improving these adverse patient characteristics, as well as improving survival following HT when compared to the use of medical therapy alone. While reversal of the HF phenotype certainly contributes to this favorable impact on outcomes, promotion of rehabilitation also plays a critical role in this process. Rehabilitation, however, is a broad term that encompasses multiple potential components, whose individual contributions to outcomes are not always apparent; as such, they may be overlooked and underappreciated. This article will define rehabilitation in the context of pediatric VAD support and review the various ways in which VAD support facilitates rehabilitation in children with chronic HF.

## What is rehabilitation?

According to the Oxford English Dictionary, “rehabilitate,” when used as a verb, is defined as “to restore to a previous condition” or “to return something to its normal or proper condition.” It is derived from the post-classical Latin word *rehabilitare*, meaning to re-establish.^9^ Thus, rehabilitation in the context of VAD can be thought of as use of the device to re-establish a patient’s former state of health prior to the development of HF. Viewed in this light, the objective of rehabilitation is not simply to stabilize a patient’s condition, but to restore the state of wellness and functional status that existed prior to the development of HF. Cardiac rehabilitation has long been recognized as an integral part of the comprehensive management of HF. When considering the essential elements of cardiac rehabilitation, it is useful to refer to the most recent practice guideline for the management of HF in adults from the American Heart Association, which defines cardiac rehabilitation as comprising the following elements: (1) exercise training and physical activity counseling program; (2) dietary recommendations; (3) psychosocial support; and (4) education regarding the importance of medical adherence.^10^ For the purposes of this review, the first 3 elements (exercise/physical activity, nutrition, and psychosocial support) will be evaluated in detail.

### Exercise training and physical activity counseling

Inactivity is a hallmark of advanced stages of HF, and major grading systems for the assessment of HF severity such as the New York Heart Association and Ross classifications use the severity of activity impairment as the primary differentiator among various classes of HF severity. While inadequate cardiac output is certainly a significant driver of inactivity, other factors such as sarcopenia, poor nutrition, respiratory insufficiency, barriers to movement imposed by medical care (i.e., central venous catheters, feeding tubes, etc.), inadequate social support, and a perception that exercise may be dangerous in HF all contribute to diminished mobility and exercise in patients with HF.

With a VAD in place and functioning as intended, normal cardiac output is restored, allowing for reversal of the HF phenotype and normalization of the end-organ dysfunction that characterizes advanced stages of chronic HF. There is a large body of evidence in adult populations with VAD demonstrating the benefit and effectiveness of structured exercise programs in patients on VAD support. For example, a randomized trial of adult patients with LVAD of a 6-week structured exercise program with individualized exercise prescriptions resulted in significant improvements in functional status and patient-reported health status, when compared to patients who received standard treatment that did not involve an exercise program.^11^ Another study evaluating the Medicare claims database demonstrated that adult VAD patients who received cardiac rehabilitation (which includes exercise) had lower rates of hospitalization and mortality compared to those who did not.^12^ An important finding from the latter study was the low participation rate in cardiac rehabilitation (30% for the entire cohort).

Pediatric data regarding exercise training on VAD with large cohorts of patients are sparse, but a few small single center studies have demonstrated the feasibility and benefit of structured exercise programs for children on various forms of VAD support.^13^, ^14^ Burstein and colleagues^13^ reported in detail their center’s protocol for baseline assessment of exercise capacity along with a structured exercise training program in 29 patients on VAD; paracorporeal and intracorporeal devices were included in the study cohort. After initial postoperative recovery and achievement of independent ambulation, patients were subjected to a cardiopulmonary exercise test using a cycle ergometer. The results of this testing allowed for the development of an individualized exercise prescription incorporating aerobic and isometric activities; this prescription involved 2 to 3 sessions per week, each lasting 30 to 60 minutes. Sessions were offered to both inpatients and outpatients. No adverse events or premature discontinuation of sessions occurred over a total of 864 sessions.

Outcomes data of exercise training in pediatric VAD recipients are very limited, however. One single center study evaluated 6-minute walk distances and B natriuretic peptide concentrations in patients before and after VAD implantation.^15^ Patients in this cohort did undergo exercise rehabilitation, although the specific regimens were not described. Nonetheless, the investigators found that 6-minute walk distances increased significantly in the first 90 days following VAD implantation. Another study analyzing the Pediatric Interagency Registry for Mechanical Circulatory Support (Pedimacs) found that inability to ambulate at 1 month following VAD implantation predicted mortality on device as well as failure to discharge for patients on dischargeable devices.^16^ Both of these studies highlight the importance of mobility following VAD, which is clearly modifiable and influenced by a regular regimen of exercise training.

Unfortunately, similar to what has been in observed in adult VAD populations, exercise rehabilitation in children on VAD support is underutilized. Burstein and colleagues performed a survey of participating centers in the Advanced Cardiac Therapies Improving Outcomes Network (ACTION) to understand individual center practices around exercise rehabilitation in children on VAD^17^ and found that only 38% of responding centers performed any form of exercise testing in children after VAD implantation, with the majority of testing performed being the 6-minute walk as opposed to more intensive regimens such as the treadmill or cycle ergometer. Commonly reported barriers to implementing a standardized exercise rehabilitation program in this survey included inadequate staff, lack of space, and distance required for patients/families to travel in order to undergo rehabilitation.

Several important practical considerations regarding exercise rehabilitation in children on VAD support are worthy of mention. First, issues of safety include ensuring adequate training of exercise facility staff regarding the management of VAD alarms, device-related complications (i.e., management of controller and/or power source disconnection), and the recognition and management of arrhythmias that may be potentiated by exercise. Second, driveline and cannula site management should not be overlooked, since the movement associated with exercise can place stress on the sites of driveline/cannula entry and increase the risk of infection and/or site disruption. These risks may be increased in small children with the Berlin Heart EXCOR (Berlin Heart, Berlin, Germany) device given the relatively large inflow and outflow cannulas relative to body size. At the author’s own center (Children’s Hospital of Philadelphia), significant efforts are employed to immobilize the cannulas (for paracorporeal devices) or driveline (for intracorporeal devices) as much as possible during exercise and other rehabilitation efforts through the use of harnesses, abdominal binders, and reinforced dressings. Finally, optimizing VAD settings to optimize exercise performance should be considered in patients with continuous flow devices. In a small study of pediatric VAD recipients with continuous flow devices, increasing pump speed during exercise increased exercise capacity (as measured by maximal oxygen consumption and physical working capacity) in patients with both LVAD and single ventricle VAD.^18^ However, similar findings have not been reliably replicated in adults,^19^ reflecting the heterogeneity in patient populations and multiple contributing mechanisms to diminished exercise capacity in patients on VAD support.^20^ Such factors include chronotropic incompetence, right ventricular failure, and sarcopenia. Indeed, an interesting finding in adult studies of VAD recipients is that maximal oxygen consumption may not with exercise rehabilitation despite a salutary impact on overall outcomes.^21^, ^22^

An emerging area of interest is the concept of remote monitoring of patients with VADs utilizing commercially available, consumer-focused wearable devices such as the iWatch (Apple, Cupertino, California USA) or FitBit (Google, Mountain View, California USA) to allow clinicians to understand patient activity patterns and monitor adherence to exercise prescriptions. In addition, implantable hemodynamic monitors such as the CardioMEMS (Abbott, Abbott Park, Illinois USA) permit continuous remote noninvasive monitoring of pulmonary artery pressure. The use of such devices may help close access to care gaps for patients on dischargeable devices who reside at large distances from the hospital, or may identify early changes in clinical status warranting hospital admission. However, experience with use of such devices in pediatric HF, much less as an adjunct to exercise rehabilitation in patients on VAD, is very limited in the literature.^23^ To address this knowledge gap, the ACTION network is currently enrolling patients in a wearable device project to improve physical activity in children with chronic HF.

Finally, it should be mentioned that for patients in whom a VAD is implanted as a bridge-to-HT strategy, the timing of HT listing relative to VAD implant is a matter of some debate. In older children and adolescents of adult size, HT waiting times are relatively short compared to infants and younger children.^1^ This difference can largely be attributed to the fact that pediatric patients (<18 years of age at time of listing) receive the highest priority heart transplant listing status in the US (United Network for Organ Sharing status 1A) when a VAD (of any type) is implanted and retain this listing status for as long as the VAD is in place. This is in contrast to adult patients with durable VAD, who are listed at lower priority levels. Adult-sized children with VADs in place are therefore inherently advantaged in the current US heart allocation system. Therefore, for these older pediatric populations implanted with a durable dischargeable VAD, waiting times are considerably shorter than that for adults supported similarly. In the ACTION registry report on patients supported with the HeartMate 3 (Abbott, Abbott Park, Illinois USA) continuous flow durable VAD, for example, median duration of support for patients who underwent HT was 72 days.^24^ While this relatively brief anticipated period of listing for older children may be viewed as favorable if the desired outcome is expeditious HT, a potential disadvantage of relatively short waiting times for HT is that patients may not fully benefit from the rehabilitative potential of VAD, which as previously discussed, has a positive effect on post-HT outcomes. Some centers have therefore adopted policies of not activating patients on the HT waiting list until at least 90 days have elapsed following VAD implant to ensure that patients have demonstrated an ability to rehabilitate, as well as to assess for the potential of myocardial recovery.^25^ The impact of this delayed listing strategy on post-HT outcomes are not clear in recent analyses of larger registry data, with some studies demonstrating a benefit for functional status and post-HT outcomes in VAD patients supported for >2 months^26^ while others have not^27^; the latter finding of no impact of longer time on VAD may be related to the deleterious impact of late adverse events experienced while on VAD support.

### Dietary management

As discussed previously, patients in chronic HF are often malnourished and exhibit poor oral intake.^28^ Such malnourishment has a multitude of adverse impacts, such as poor wound healing, retardation of rehabilitation, and decreased mobility. Most studies of malnourishment in pediatric patients define malnutrition as a weight-for-age Z score, height-for-age Z score, or body mass index-for-age Z score of <2, as outlined by the American Society for Parenteral and Enteral Nutrition.^29^ There are limitations to the use of weight as an assessment in children with HF owing to volume overload and the practical challenges of obtaining regular and reliable patient weights in the healthcare setting. Other measurements, such as mid-upper arm circumference, have normative values and can be used in the clinical setting as well to define malnutrition and follow response to nutritional interventions.

One study has specifically evaluated the impact of VAD on the nutritional status of children awaiting HT. Hollander and colleagues evaluated a cohort of children awaiting HT stratified by whether they underwent HT with a VAD in place or not, using height-for-age and weight-for-age Z scores as a metric of nutritional status.^30^ Malnutrition at the time of HT listing was high, affecting 70% of the cohort. Compared to patients in the medical therapy subgroup, VAD-supported patients had an increase in height-for-age and weight-for-age Z scores, with a decrease in the prevalence of moderate or severe malnutrition. These changes were not observed in the medical therapy group. A subsequent study from the same institution evaluating the prevalence of feeding disorders in children listed for HT found that VAD use was associated with renourishment (normalization of anthropometric Z scores) during the waiting period for HT.^31^

One challenge to re-establishing adequate nutrition in patients on VAD is that in the early postoperative phase, nausea and anorexia are common. Feeding intolerance is also frequently observed in infants on VAD, likely related to the fact that the inflow and outflow cannulas for paracorporeal devices are relatively large compared to patient size and may traverse the peritoneum. Although less commonly encountered than with earlier generations of devices, pancreatitis has been reported following VAD implantation and should be considered in children with unexplained feeding intolerance after implantation. In addition, gastrointestinal bleeding is a known complication of patients on VAD, which can cause significant feeding intolerance. Gastrointestinal bleeding affects 4–8% of children on VAD support, with higher rates noted in patients on paracorporeal continuous and paracorporeal pulsatile devices compared to intracorporeal devices.^32^ The most common cause of gastrointestinal bleeding in pediatric VAD patients is related to the need for systemic anticoagulation and antiplatelet therapy, with such regimens usually being more intense in patients supported with paracorporeal devices. However, gastrointestinal bleeding may also occur in patients with intracorporeal continuous flow devices owing to decreased von Willebrand factor activity mediated by shear stress; however, the risk of acquired von Willebrand deficiency and associated gastrointestinal bleeding is lower in newer generation continuous flow VADs (HeartMate 3) compared to earlier generation pumps (HeartMate 2, HeartWare HVAD) owing to unique pump characteristics such as magnetic levitation and the presence of an artificial pulse.^33^ Further complicating the re-establishment of adequate nutrition following VAD is the fact that surgical feeding tubes (i.e., gastrostomy) may be infeasible given the proximity of VAD drivelines and/or cannulas; this is particularly the case for infants due to their small size. Nevertheless, successful feeding and renourishment are possible in patients on VAD and involves the coordinated involvement of a multidisciplinary team of physicians, nurses, nutritionists, and rehabilitation specialists. For patients with significant feeding intolerance following VAD implant, early involvement of the multidisciplinary team with specialty input from pediatric gastroenterologists and nutritionists is essential to establish successful nutrition. For example, gastroenterology consultation may be useful in determining optimal medical therapy as well as feeding route (gastric, post-pyloric, etc.) for patients with feeding intolerance. The nutritionist’s role is essential in assessing the patient’s diet, current nutritional status, and nutritional deficiencies, with recommendations for specific form of nutrition based upon this assessment. It should also be noted that achieving successful nutrition in a chronically ill child on VAD can be fatiguing and frustrating for the patient’s caregivers, so consistent support at the bedside from the nursing and rehabilitation teams is crucial for long-term success. For reference, the ACTION network has published a harmonization protocol for nutritional management on VAD which is available at www.myactioneducation.org.

### Psychological aspects of pediatric VAD support

Chronic HF has significant impacts on multiple dimensions of well-being, including mental health, coping, relationships with others, and QOL. For example, children with HF have impaired QOL, functional status, and overall psychological well-being, which can be improved by HT.^31^, ^32^, ^33^, ^34^, ^35^, ^36^ The limited data available suggest that QOL improves after VAD implantation in children, but not necessarily across all domains.^37^, ^38^, ^39^, ^40^ For example, in the largest series of QOL outcomes in children supported with VAD to date,^37^ 82 children had QOL data available from the Pedimacs registry; total and psychosocial QOL scores increased post-VAD compared to pre-VAD assessment, but the physical component of QOL did not change after VAD implantation. This finding argues for increasing physical rehabilitation among children with VAD.

Psychiatric disorders, such as depression, anxiety, and adjustment disorder, are common in children with advanced HF being considered for VAD and HT. Using a linkage between the Scientific Registry of Transplant Recipients and Pediatric Health Information System databases, psychiatric disorders were identified in nearly 40% of children supported with a VAD at the time of HT,^41^ with VAD support being an independent risk factor for such disorders. Although data are lacking in the pediatric population, suicidal behavior (ideation and suicidal acts) appear to occur at significantly higher rates than the general population in adult VAD recipients.^42^, ^43^ Addressing disorders of mood in children on VAD can be challenging, as most clinicians directly caring for such patients and managing the device (cardiologists, surgeons) are usually ill-equipped to diagnose and manage mental health conditions. However, the recognition and effective management of these conditions is crucial, not only to improve the well-being and daily life experience of patients on VAD, but also because the presence of psychiatric disorders has been shown to have a deleterious impact on adherence to the medical regimen following HT.^44^ Furthermore, mood disorders in patients on VAD may limit the effectiveness of exercise rehabilitation. For example, the presence of depression in adult VAD patients impeded effective exercise rehabilitation as evidenced by lower improvement in maximal oxygen consumption in a small study.^45^ For these reasons, formal and age-appropriate mental and behavioral health assessment is recommended for all patients undergoing VAD support, with regular follow-up after VAD implant as well.

It has long been recognized that psychological well-being and adequacy of social support structures are necessary to the long-term success of HT, with inadequate social support remaining a relative contraindication to HT in the current era.^46^ For patients supported on VAD prior to HT, the pre-HT waiting period in which physical rehabilitation is prioritized affords ample opportunities for the care team to ensure the psychological and social well-being of the patient and his/her caregivers. Psychosocial considerations in pediatric VAD recipients were recently reviewed systematically.^47^ Several themes were identified in this review, particularly the increased stress and difficulty coping experienced both by VAD recipients and their caregivers. In addition, several periods of increased psychosocial risk were identified: (1) immediately after VAD placement; (2) early following discharge; and (3) for patients on long-term VAD therapy. A recent survey through the ACTION network indicates that psychosocial services available to patients and families are relatively lacking,^48^ particularly in the domains of neurodevelopmental care and support for parents, identifying a critical opportunity for improvement in the comprehensive care of VAD patients, their families, and caregivers.

### Rehabilitation efforts at Children’s Hospital of Philadelphia

Considerable variability exists among pediatric VAD programs with respect to the specific protocols and resources directed toward the rehabilitation of patients on VAD, which are often structured based on program size, needs, and capacities and reflect the individual characteristics of each institution. This author will provide a brief overview of rehabilitation initiatives at his own institution in order to provide the reader with a general perspective, which also may be useful to centers that are developing their own VAD programs. The first VAD was implanted at Children’s Hospital of Philadelphia in 1998. Since that time, over 150 patients have been implanted with a VAD, with current annual implant volume of approximately 20 patients per year. Over the lifespan of the VAD program, considerable evolution has occurred in order to provide the most comprehensive care to patients, including rehabilitation services. In late 2023, we inaugurated an Advanced Cardiac Therapies (ACT) program at our institution in order to optimize care for children with chronic HF, with an emphasis on improving outcomes of children on VAD through coordination of care, offering VAD therapy to patient populations previously underserved by VAD, and enhanced rehabilitation services.

A major component of the ACT Program is intensive rehabilitation for inpatients on VAD; this program has direct involvement of multiple specialty teams distinct from the primary medical team including physical therapy, occupational therapy, speech therapy, exercise physiology, cardiac perfusion, and physical medicine and rehabilitation. Once eligible patients are determined to be medically appropriate for the intensive rehabilitation (typically within 1 week following implant), they are entered into an “intensive phase” which involves therapy sessions 5–7 days/week with up to 3 hours of therapy daily. This intensive phase is transitioned to a “conditioning phase” once patients have achieved their baseline status or have reached a plateau in skills development during the intensive phase; during this conditioning phase patients undergo therapy sessions 3 times weekly. Within the intensive phase is a “developmental intensive” phase for infants, which has additional efforts geared toward the evaluation and achievement of age-appropriate developmental milestones.

At our institution, all patients being considered for VAD undergo evaluation by the heart transplant team psychologist, with consults for formal neuropsychiatric testing and psychiatry placed if indicated by the psychology evaluation. For school-age children hospitalized with non-dischargeable devices, in-person schooling is provided. For those patients with a dischargeable device in place, coordination between the VAD team and the patient’s local school occurs in order to allow patients to attend in-person school in their community, with educational modifications as necessary through individual educational plans. Coordination with the local school district involves in-person visits by the VAD team to educate teachers and other school staff on the device, alarm management, and emergency management. Finally, parent/caregiver support for children hospitalized on VAD is provided with the availability of parent support groups, in-hospital amenities, social work support, and referral to mental health providers for caregivers as needed. For patients who can be discharged on VAD, the case management team coordinates all aspects of discharge, including provision of supplies, determining whether home nursing support is feasible, and arrangement of follow-up visits. Extensive teaching to caregivers on home management of the VAD is provided by the VAD nursing coordinator. Patients and families on dischargeable VADs are provided with a telephone number that contacts the on-call VAD attending physician 24 hours/day, 7 days/week for any concerns.

For patients implanted with dischargeable devices, sessions in the exercise laboratory are offered to patients during regular clinic visits and weekly sessions are made available to those who are within a reasonable driving distance to the institution. Patients are offered an individualized exercise prescription tailored to their device, physiology, and overall medical condition. Table 2, Table 3 present the components of exercise rehabilitation and intensive rehabilitation programs, respectively, at Children’s Hospital of Philadelphia.

**Table 2:** Exercise Rehabilitation Program for Ventricular Assist Device Patients at Children’s Hospital of Philadelphia

• Early ambulation and mobility (within 2 days of initial postoperative extubation) • Baseline cardiopulmonary exercise test (cycle ergometer) if following criteria are in place: o >14 days from implant o >6 years of age o Off vasoactive infusions o No active hematologic concerns o No wound infection concerns or poor wound healing o No active myocarditis o No arrhythmia burden refractory to medical therapy • Baseline cardiopulmonary exercise test characteristics o 3 minutes unloaded warm-up pedaling period o Ramped increase in work rate to achieve predicted maximal work rate within 10−12 minutes o Assessment of metabolic and ventilatory data using metabolic cart o Exercise parameters measured: heart rate, VAD flow, work rate, maximal oxygen consumption (VO_2_), pulsatility index, oxygen saturation at rest, anaerobic threshold, and maximal exercise o Respiratory exchange ratio ≥1.1 defines maximal exercise • Chronic exercise rehabilitation program components (individualized based on patient characteristics) o Moderate aerobic exercise with heart rate at ventilatory aerobic threshold o Resistance exercises for upper extremities, lower extremities, torso (9 exercises in total, with 2 sets of 15 repetitions) o 2−3 sessions per week, with each session 30−60 minutes • Safety considerations o Limited to moderate exercise intensity o Prolonged gradual warm-up and cool-down periods (10−15 minutes each) o Avoidance of breath-hold or Valsalva maneuver o Stabilization of VAD driveline/cannulas o Avoidance of running, rowing, swimming, abdominal exercises, bilateral arm extension above the head o Close supervision by practitioners familiar with VAD technology

VAD, ventricular assist devices.

Refer to reference Burstein et al.^13^ for details.

**Table 3:** Intensive Rehabilitation Program for Inpatient Ventricular Assist Device Patients at Children’s Hospital of Philadelphia

• Acute Phase o Started as soon as clinical stability is achieved following VAD implant o Therapies offered twice weekly • Intensive Phase o Offered to patients >12 months of age once clinically determined to be appropriate for increased rehabilitation frequency o Therapies offered 5−7 days per week, up to 3 hours per day o Infants 4–12 months of age receive “developmental intensive” therapy to provide developmental skills during a crucial window of development • Conditioning Phase o Begins when therapy team determines that patient is at baseline level of conditioning or has plateaued in skill development and continues through the duration of VAD

VAD, ventricular assist devices.

Therapies include physical therapy, speech therapy, occupational therapy, and exercise rehabilitation. Therapies are provided “in place” when appropriate. Maintenance of a consistent schedule of therapies is crucial.

## Conclusions

Rehabilitation is crucial to optimizing outcomes on VAD and includes exercise, nutritional, and psychosocial domains. The benefits of rehabilitation translate into improved outcomes following HT (for those patients in whom a VAD is placed as a bridge to transplant), and also improve QOL for pediatric VAD recipients. However, opportunities for improvement include increasing the availability of exercise rehabilitation to children on VAD and expansion of psychosocial support to VAD patients and their families/caregivers. Collaborative efforts through multicenter networks such as ACTION are crucial for the dissemination of knowledge and experience necessary for the optimal care of this complex patient population.

## Declaration of Competing Interest

The authors declare that they have no known competing financial interests or personal relationships that could have appeared to influence the work reported in this paper.
